# A Rare Occurrence of a Huge Ovarian Thecoma in a Menopausal Patient, 25 Years After Abdominal Hysterectomy and Right Salpingo-Oophorectomy: A Case Report and Mini-Review of the Literature

**DOI:** 10.7759/cureus.73258

**Published:** 2024-11-07

**Authors:** Anna Thanasa, Efthymia Thanasa, Ioannis-Rafail Antoniou, Gerasimos Kontogeorgis, Emmanouil M Xydias, Apostolos C Ziogas, Ioannis Thanasas

**Affiliations:** 1 Department of Health Sciences, Medical School, Aristotle University of Thessaloniki, Thessaloniki, GRC; 2 Department of Obstetrics and Gynecology, General Hospital of Trikala, Trikala, GRC; 3 Department of Obstetrics and Gynecology, EmbryoClinic IVF, Thessaloniki, GRC; 4 Department of Obstetrics and Gynecology, University of Thessaly, Larissa, GRC

**Keywords:** case report, chronic pelvic pain, computed tomography, magnetic resonance imaging, ovarian thecoma, surgical treatment, transvaginal ultrasound

## Abstract

Our case involves a 68-year-old postmenopausal patient with a history of total abdominal hysterectomy and right salpingo-oophorectomy performed 25 years ago. The patient presented with chronic pelvic pain for a gynecological examination. Clinically, a large painless pelvic mass was palpable, likely originating from the preserved left ovary. Transvaginal ultrasonography, computed tomography, and magnetic resonance imaging supported the clinical suspicion, and an exploratory laparotomy was subsequently performed. Intraoperatively, a solid, neoplastic, exophytic ovarian tumor was discovered, occupying the entire pelvis. The tumor, along with the ovary and the corresponding fallopian tube, was surgically removed. Histological examination of the specimen confirmed the diagnosis of an ovarian thecoma. Following a four-day hospitalization with an uneventful postoperative course, the patient was discharged from our clinic. Three months later, she reported complete relief from chronic pelvic pain. This report of the case also includes a brief literature review, emphasizing the preoperative diagnostic challenges of this rare clinical entity and the importance of regular gynecological check-ups for women who have undergone total hysterectomy with ovarian preservation.

## Introduction

Chronic pelvic pain in women is defined as persistent, non-menstrual pain located in the pelvis, lasting for more than six months [1]. It is a significant social issue, estimated to affect up to 20% of women [2]. Chronic pelvic pain accounts for approximately 40% of laparoscopies and 12% of hysterectomies performed annually in the United States, despite the fact that in 80% of cases, its etiology is not gynecologic [3]. The etiopathogenesis of chronic pelvic pain is typically non-specific, as it is a multifactorial disorder. It is often linked to dysfunction of the female reproductive system, including conditions such as endometriosis, adenomyosis, uterine leiomyomas, ovarian cystic neoplasms, thecomas, fibromas, and ovarian fibrothecomas. Less commonly, it may be associated with disorders of the urinary, gastrointestinal, or pelvic nervous systems, all of which can significantly impact the quality of life [4,5].

Ovarian thecomas, fibromas, and fibrothecomas are rare, non-cancerous sex cord/stromal tumors [6]. Sex cord/stromal tumors account for less than 5% of all ovarian neoplasms [7]. Thecomas are rare benign tumors of the ovarian stroma that can secrete estrogen, androgens, or a combination of both. They are estimated to represent less than 1% of all benign ovarian neoplasms and typically occur in premenopausal and postmenopausal women aged 50-60 years [8]. The occurrence of thecomas in childhood and adolescence is exceedingly rare [9]. Ovarian thecomas are usually unilateral, with tumor sizes ranging from 5 cm to 10 cm, and bilateral involvement in only 3% of cases [10]. In extremely rare instances, ovarian thecomas have been reported during pregnancy [7].

This paper presents the case of a 68-year-old woman diagnosed with a large ovarian thecoma, 25 years after undergoing a total abdominal hysterectomy with unilateral salpingo-oophorectomy. The case underscores the preoperative diagnostic challenges associated with this rare ovarian neoplasm. Additionally, it highlights the importance of regular pelvic examinations in women who have undergone total hysterectomy with ovarian preservation, irrespective of the presence or absence of pelvic symptoms such as chronic pelvic pain.

## Case presentation

A 68-year-old patient, with a history of two vaginal deliveries and an abdominal total hysterectomy with right salpingo-oophorectomy performed 25 years ago due to uterine leiomyomas and menometrorrhagia, was referred from the gastroenterology department to the gynecology department for further investigation of chronic pelvic pain. The onset of the pain dated back approximately 18 months and was described as mild, deep, constant, and occurring daily. A colonoscopy revealed no abnormal findings and clinical and imaging examinations of the urinary tract showed no lesions affecting the kidneys, ureters, or bladder. The patient's medical history included well-controlled arterial hypertension and hypothyroidism. Her family history was unremarkable.

**Figure 1:**
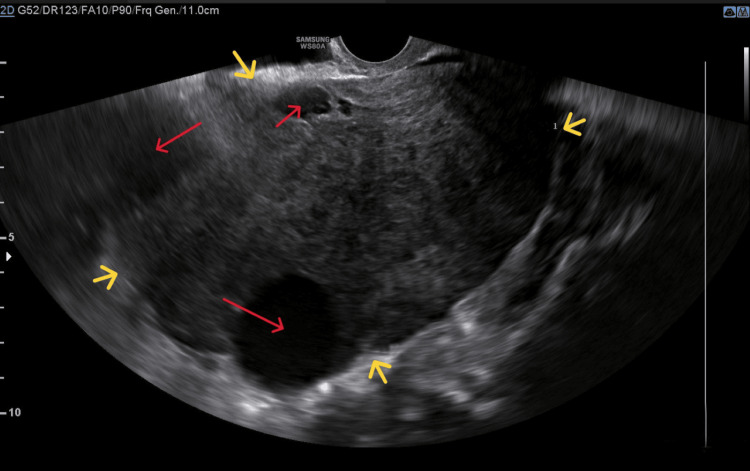
Transvaginal ultrasound imaging of the ovarian thecoma A well-defined pelvic mass (yellow arrows) is visible, primarily solid with some cystic elements (red arrows)

During the vaginal examination, a large, painless pelvic mass was detected, occupying the pouch of Douglas. Transvaginal ultrasound revealed a well-defined echogenic mass with both solid and cystic components, filling the entire pelvis (Figure 1).

**Figure 2:**
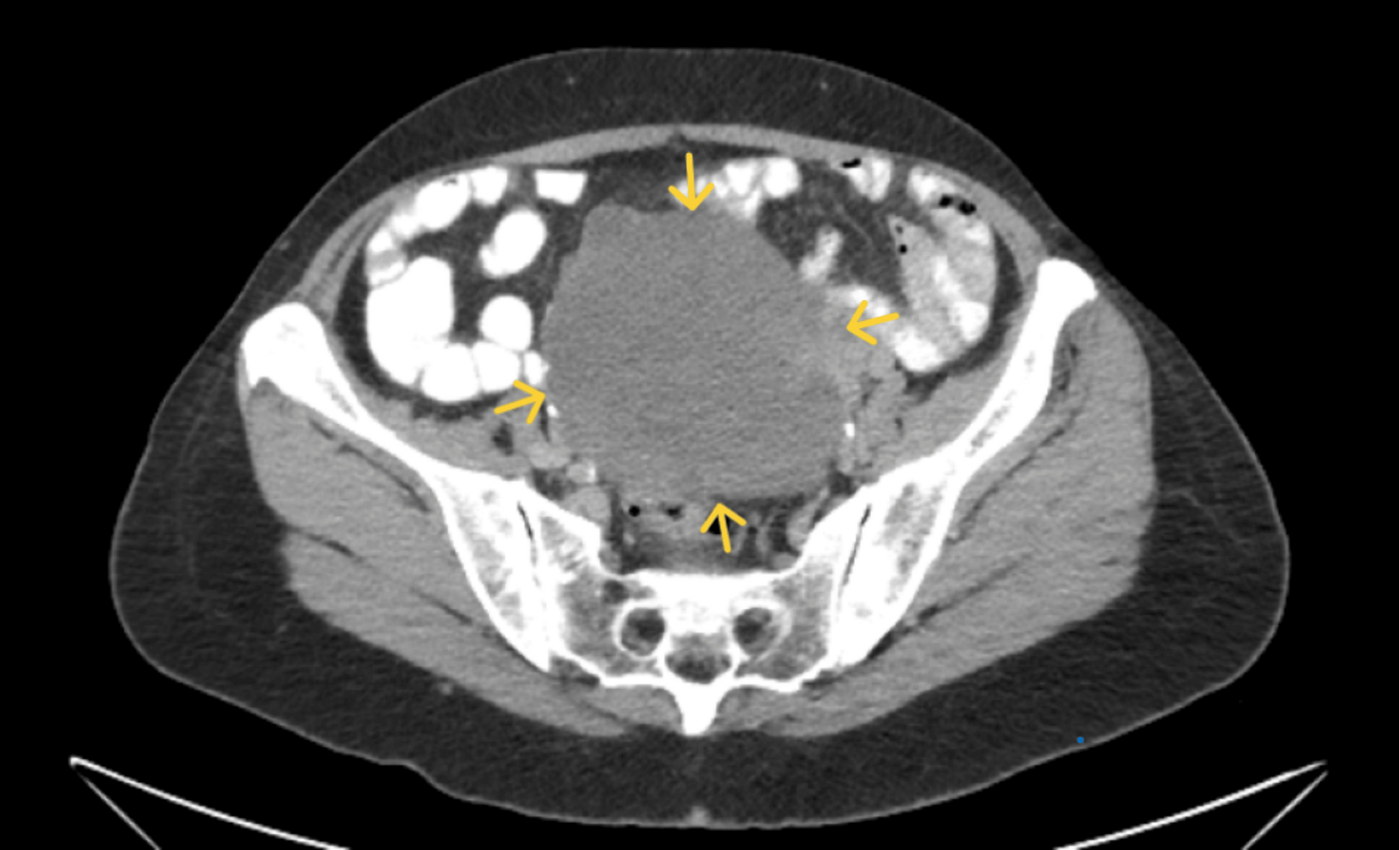
CT imaging of the ovarian thecoma A large mass with heterogeneous enhancement is evident (yellow arrows), originating from the left ovary and occupying the Douglas space

A computed tomography (CT) scan confirmed a large lesion with cystic and solid elements, showing heterogeneous enhancement, which appeared to arise from the left ovary. A small collection of free fluid was noted in the Douglas space (Figure 2).

**Figure 3:**
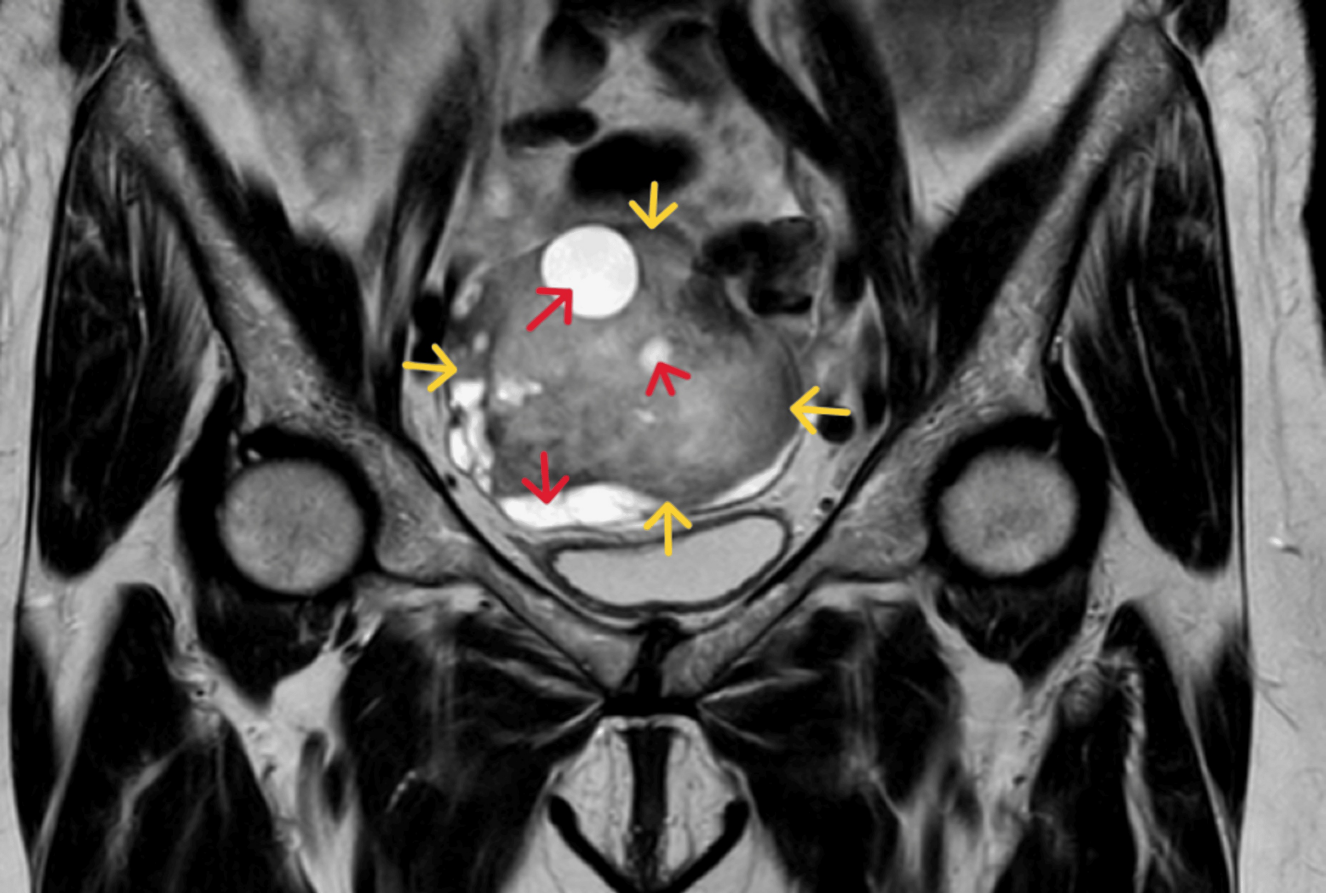
MRI of the ovarian thecoma The lesion shows low-intensity signal in both T1 and T2 sequences (yellow arrows) with signs of cystic degeneration (red arrows)

Magnetic resonance imaging (MRI) demonstrated a lesion with low signal intensity on the T1 sequence. On the T2 sequence, the lesion showed low signal intensity and signs of cystic degeneration. It appeared to be homogeneously enhanced, with no evidence of abnormal lymph nodes in the surrounding area (Figure 3).

Laboratory tests (Table 1) were conducted to complete the preoperative assessment, and surgical management by laparotomy was decided.

**Table 1:** Laboratory tests as part of the patient's preoperative check-up Ht: hematocrit; Hb: hemoglobin; WBC: white blood cells; NEUT: neutral; Glu: glucose; U: urea; Cr: creatinine; CEA: carcinoembryonic antigen; CA: cancer antigen

Laboratory Tests	Preoperative Values	Laboratory Reference Values
Ht	41%	37.7 – 49.7%
Hb	13.1 gr/dl	11.8 – 17.8 gr/dl
WBCc	7.1x10^3^/ml	4 – 10.8 x10^3^/ml
NEUT	63%	40 – 75%
Glu	107 mg/dl	75 – 115 mg/dl
U	51 mg/dl	10 – 50 mg/dl
Cr	0.9 mg/dl	0.40 – 1.10 mg/dl
CEA	3.45 ng/mL	< 5 ng/mL
CA 125	17.8 U/mL	≤ 35 U/mL
CA 15-3	14.1 U/mL	0.0 – 31.3 U/mL
CA 19-9	13.4 U/mL	0.0 – 37 U/mL

**Figure 4:**
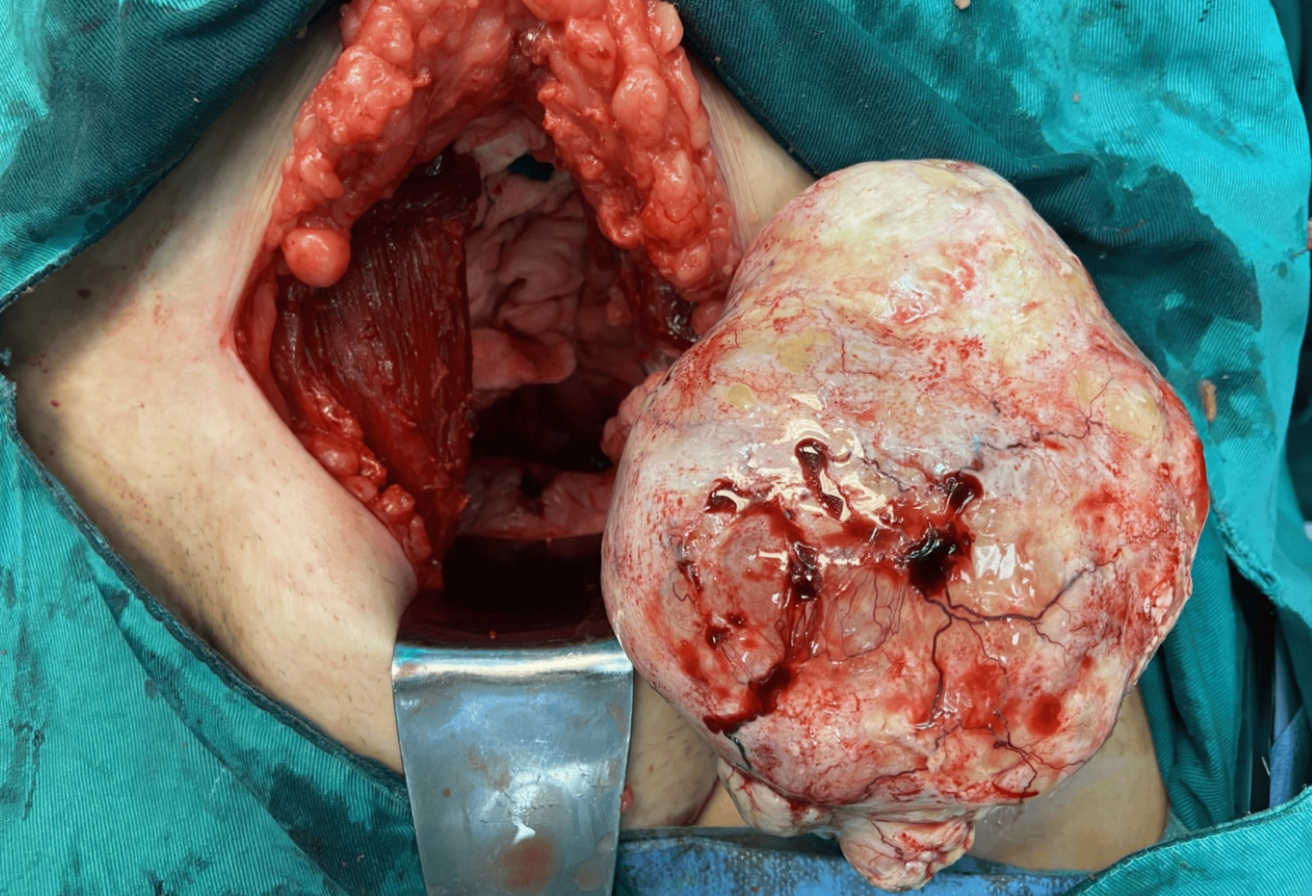
Intraoperative imaging of the ovarian thecoma A solid ovarian mass is observed, emerging from the pelvis without adhering to adjacent tissues

Intraoperatively, a large ovarian mass, predominantly solid with some cystic elements, was identified, measuring approximately 15 cm in diameter. The mass was wedged in the pelvis but showed no infiltration of adjacent tissues (Figure 4).

**Figure 5:**
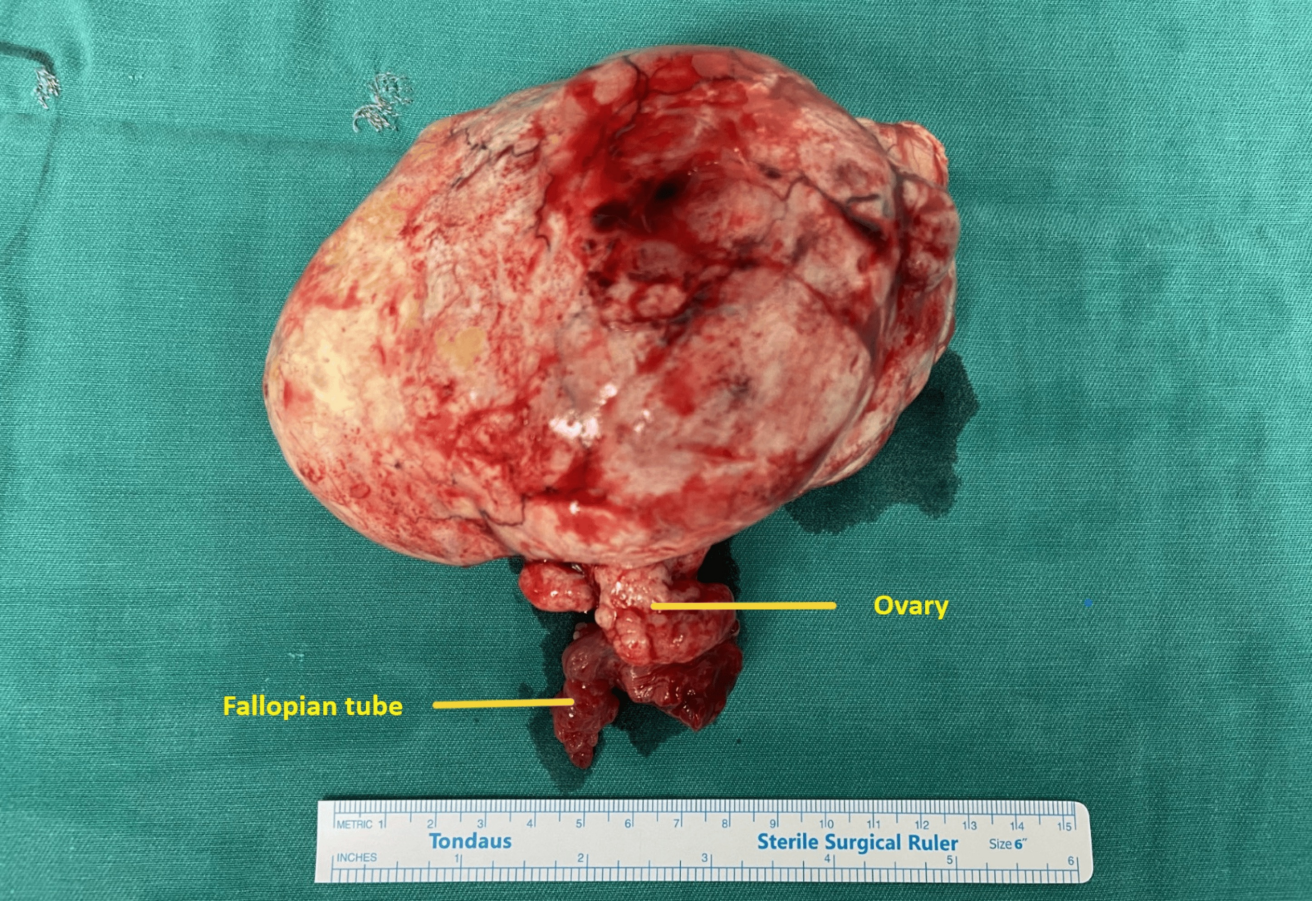
Operative specimen of the ovarian thecoma A large, oval, exophytic ovarian mass (ovarian thecoma) is clearly visible

After ligation of the left infundibulopelvic ligament, the neoplastic ovarian tumor was excised along with the left ovary and fallopian tube (Figure 5).

**Figure 6:**
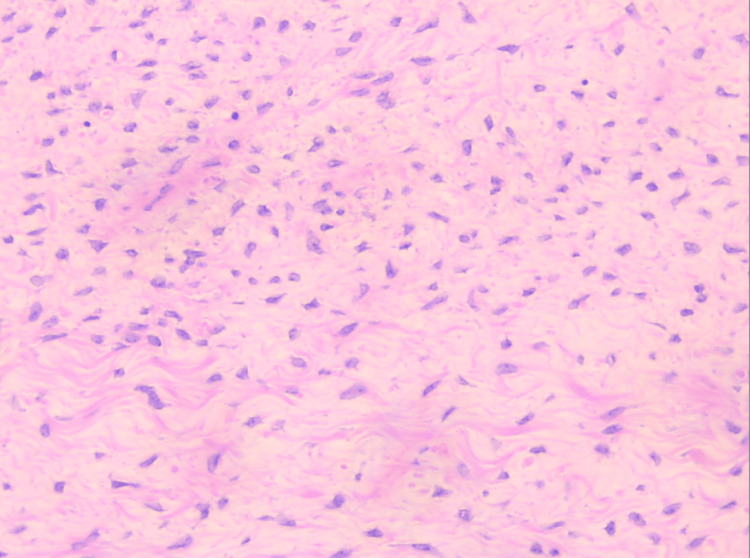
Histological image of the ovarian thecoma Depiction of uniform cells with pale cytoplasm, showing no nuclear atypia and no mitoses (Hematoxylin-Eosin stain, x20 magnification)

Histological examination confirmed the diagnosis of an ovarian thecoma. The specimen was an oval mass weighing 680 grams with a smooth, whitish outer surface. Cross-sections revealed cysts containing serous fluid and solid areas with a bundled appearance, whitish color, fibroelastic texture, and focal cystic degeneration, with no evidence of atypia or mitoses (Figure 6).

**Figure 7:**
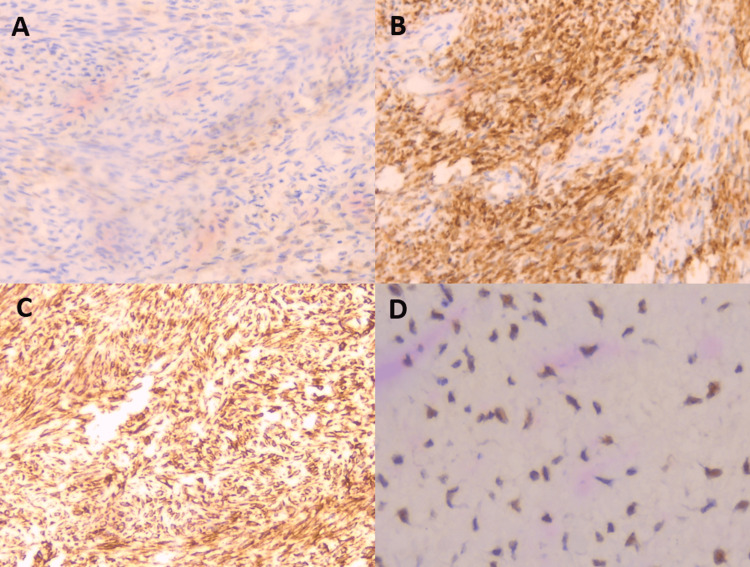
Immunohistochemical images of the ovarian thecoma (A) Positive staining for calretinin; (B) Positive staining for inhibin; (C) Positive staining for vimentin; (D) Positive staining for Wilms' tumor protein

Immunohistochemical analysis demonstrated strong positivity for inhibin (+++), calretinin (+++), Wilms' Tumor Protein (WT1) (+++), and vimentin (+++) (Figure 7).

After a four-day hospitalization with an uneventful postoperative recovery, the patient was discharged. Three months later, she reported complete relief from her chronic pelvic pain.

## Discussion

The etiopathogenetic mechanisms of sex cord/stromal ovarian neoplasms have not yet been fully elucidated. Sex cord/stromal ovarian tumors are rare ovarian neoplasms that include various tumor subtypes with diverse histological features and biological behaviors [11]. Ovarian thecomas are classified as a subtype of stromal tumors according to the World Health Organization classification, revised in 2014 [12]. Ovarian thecomas consist of stromal cells containing lipids (theca cells), which surround ovarian follicles and typically exhibit estrogenic activity [13]. Additionally, genetic syndromes such as Peutz-Jeghers syndrome and DICER1 syndrome are thought to be associated with the pathogenesis of ovarian thecomas [14].

The clinical manifestations of ovarian thecomas are non-specific, making preoperative diagnosis challenging [15]. Most patients with ovarian thecoma are asymptomatic, particularly when the tumors are small in size [16]. Symptoms such as abdominal distention, vague abdominal discomfort, and chronic pelvic pain, as seen in our patient, are typically associated with larger thecomas [17]. In addition, ovarian thecomas frequently cause endocrine disorders due to hormone secretion. The secretion of androgenic hormones can result in oligomenorrhea and the gradual development of secondary male characteristics in women, such as excessive hair growth, facial acne, and clitoral hypertrophy [18]. Rarely, in postmenopausal women, ovarian thecomas can cause endometrial hypertrophy and vaginal bleeding, making it imperative to differentiate them from uterine malignancies [19]. In very rare, isolated cases, ovarian thecoma may also be associated with Meigs syndrome [20]. The presence of ascites is typically linked to larger tumors, regardless of cancer antigen 125 (CA-125) levels in the blood serum [21]. Additionally, in extremely rare instances, thecomas may occur during pregnancy, with the first clinical manifestation being acute abdominal pain caused by pedicle torsion [7]. In our patient, the diagnosis of ovarian thecoma was delayed. The patient’s history of hysterectomy likely hindered regular gynecological screenings, resulting in a missed diagnosis during the asymptomatic phase. The patient presented for examination at the gynecological clinic when the ovarian thecoma had reached a larger size, causing chronic pelvic pain due to increased pelvic pressure.

The use of modern imaging modalities plays a crucial role in the preoperative diagnosis of ovarian thecomas. Transvaginal ultrasound and Doppler ultrasound imaging of the ovaries are first-line imaging examinations. The typical sonographic features of ovarian thecomas include hypoechoic solid masses with well-defined borders and the presence of striated shadows, with minimal flow signals on Doppler ultrasonography [16,22]. MRI is a highly useful diagnostic tool for the female reproductive system and is thought to significantly improve the accuracy of preoperative diagnosis of ovarian thecomas [23]. In 2024, Zheng et al. highlighted the utility of MRI in the differential diagnosis of benign thecomas/fibromas from solid malignant ovarian neoplasms in their study [24]. Although CT cannot replace the importance of evaluating ovarian tumors with MRI, it can still be a useful tool for diagnosing ovarian thecomas/fibromas. The compact consistency of the tumor, its unilateral location, and the absence of enlarged lymph nodes or peritoneal metastases are characteristic CT findings that aid in the early diagnosis of ovarian thecoma/fibroma. These findings help reduce unnecessary referrals of patients to tertiary oncology centers, alleviating the stress that can arise from misdiagnosis of ovarian malignancy [25]. In our patient, the imaging results indicated the benign nature of the pelvic mass, which influenced our decision to proceed with laparotomy at our hospital rather than referring the patient to a tertiary gynecological oncology center. This approach spared the patient unnecessary stress and also helped avoid additional hospitalization costs.

Surgery remains the cornerstone in the treatment of ovarian thecomas. A conservative approach (tumor resection or unilateral salpingo-oophorectomy) is preferred in young women, where fertility preservation for future pregnancy is a primary goal. In older patients, more radical procedures, such as bilateral salpingo-oophorectomy with or without hysterectomy, depending on the patient’s overall condition, are generally the recommended treatment options [6]. In our patient, the only viable treatment was the surgical removal of the thecoma along with the corresponding ovary and fallopian tube. Of course, confirmation of the diagnosis of ovarian thecoma requires a histological examination of the surgical specimen. Histologically, thecomas are characterized by spindle-shaped, oval, or round cells with varying amounts of collagen and a smaller proportion of theca cells [26]. Additionally, microscopic pathological findings such as hyaline plaques, nodular growth, calcification, and keloid-like sclerosis are often observed in these neoplasms. In about 40% of cases, features of fibroma are also present. Although degenerative atypia is rarely seen, it is essential to differentiate ovarian thecomas from other stromal ovarian tumors, such as sclerosing stromal tumors, microcystic stromal tumors, steroid cell tumors, and adult-type granulosa cell tumors of the ovary [26]. The prognosis of ovarian thecomas is generally favorable [27].

## Conclusions

Thecomas are rare benign ovarian tumors. The occurrence of a large ovarian thecoma many years after a hysterectomy with unilateral salpingo-oophorectomy, as seen in our patient, is considered extremely rare. For patients with preserved ovarian tissue, regular pelvic imaging is essential, as it plays a crucial role in the early detection of such neoplasms. Surgical intervention remains the primary treatment for ovarian thecomas, emphasizing the importance of vigilant monitoring in these cases.
